# Do We Need to Personalize Renal Function Assessment in the Stratification of Patients Undergoing Cardiac Surgery?

**DOI:** 10.5935/abc.20170129

**Published:** 2017-10

**Authors:** Camila P. S. Arthur, Omar A. V. Mejia, Diogo Osternack, Marcelo Arruda Nakazone, Maxim Goncharov, Luiz A. F. Lisboa, Luís A. O. Dallan, Pablo M. A. Pomerantzeff, Fabio B. Jatene, Grupo de Estudo REPLICCAR

**Affiliations:** 1 Instituto do Coração (InCor) do Hospital das Clínicas da Faculdade de Medicina da Universidade de São Paulo (HCFMUSP), São Paulo, SP - Brazil; 2 Hospital de Base de São José do Rio Preto, São José do Rio Preto, SP - Brazil

**Keywords:** Renal Insufficiency/prevention & control, Myocardial Revascularization, Hospital Mortality, Creatinine/analysis, Indicators of Morbidity and Mortality, Risk Factors

## Abstract

**Background:**

Renal dysfunction is an independent predictor of morbidity and mortality in cardiac surgery. For a better assessment of renal function, calculation of creatinine clearance (CC) may be necessary.

**Objective:**

To objectively evaluate whether CC is a better risk predictor than serum creatinine (SC) in patients undergoing cardiac surgery.

**Methods:**

Analysis of 3,285 patients registered in a prospective, consecutive and mandatory manner in the Sao Paulo Registry of Cardiovascular Surgery (REPLICCAR) between November 2013 and January 2015. Values of SC, CC (Cockcroft-Gault) and EuroSCORE II were obtained. Association analysis of SC and CC with morbidity and mortality was performed by calibration and discrimination tests. Independent multivariate models with SC and CC were generated by multiple logistic regression to predict morbidity and mortality following cardiac surgery.

**Results:**

Despite the association between SC and mortality, it did not calibrate properly the risk groups. There was an association between CC and mortality with good calibration of risk groups. In mortality risk prediction, SC was uncalibrated with values > 1.35 mg /dL (p < 0.001). The ROC curve showed that CC is better than SC in predicting both morbidity and mortality risk. In the multivariate model without CC, SC was the only predictor of morbidity, whereas in the model without SC, CC was not only a mortality predictor, but also the only morbidity predictor.

**Conclusion:**

Compared with SC, CC is a better parameter of renal function in risk stratification of patients undergoing cardiac surgery.

## Introduction

Cost-effectiveness analysis in cardiac surgery reveals the impact of complication prevention and incorporation of new technologies in health system.^1^ High rates of complications and hospital mortality have been reported in patients with renal dysfunction who undergo myocardial revascularization surgery.^2^ Therefore, a more reliable, individualized assessment of renal function may lead to better optimization and allocation of resources that may help physicians and patients choose the best time and type of treatment.

In this context, several studies have shown a direct correlation of preoperative renal failure with morbidity and mortality following cardiac surgery.^3,4^ For a better estimate of kidney failure degree, current risk scores, such as EuroSCORE II, have included creatinine clearance (CC) calculation.^5-7^ However, EuroSCORE II has been shown to become more complex and flawed when adapted to current lines of work, as revealed by internal validation.^8,9^ For this reason, we have concerns relating to how to choose international scores and more and more complex models.

To estimate mortality risk, Brazilian models include serum creatinine (SC) values only, even as categorical variable.^10,11^ Hence, EuroSCORE II, recently validated in Brazil,^12^ includes CC levels as a predictive variable, aiming to improve the performance of the original version of EuroSCORE.^13^ However, pitfalls in calibration tests of the instrument may be related to inaccurate measurements of some variables in our settings. In light of this, and due to the higher complexity of estimating CC as compared with SC for physicians and other healthcare professionals, the real need for estimating this parameter is questionable. Unfortunately, to our knowledge, there are no studies available on the impact of CC *versus* SC on morbidity and mortality after cardiac surgery.

In light of this gap in the literature, the aim of our study was to objectively assess the importance of CC *versus* SC in the stratification of patients undergoing cardiac surgery in a prospective, multicentric, mandatory registry of patients undergoing cardiac surgery in the state of Sao Paulo, Brazil.^14^

## Methods

### Sample

Cross-sectional study based on Sao Paulo Registry of Cardiovascular Surgery (REPLICCAR), performed at Heart Institute (InCor) of the General Hospital of the University of Sao Paulo Medical School. All patients who consecutively underwent emergency coronary and/or valve surgery in 10 hospitals in the state of Sao Paulo in the period from November 2013 to January 2015 were included in the analysis. Before the start of the study, the presence of SC, CC and EuroSCORE II in all patients was confirmed. The sample should have included a minimum of 100 events for statistical significance; the study was started with 224 deaths and 263 morbidities registered.

#### Inclusion and exclusion criteria

Inclusion criteria:

All patients aged ≥ 18 years, who underwent elective surgery during the pre-established period for:

- Valve surgery (substitution or plastic surgery);
- Myocardial revascularization surgery (MRS) (with or without extracorporeal circulation)
- Combined surgery (MRS and valve surgery).

Exclusion criteria:

Other types of surgeries performed in combination with valve and/or MRS.

### Data collection, definition and organization

Collected data are fed in to REPLICCAR by a trained person in each of the 10 centers participating in the project. Data were inserted online to the website *bdcardio.incor.usp.br* by username and password, into four different interfaces: preoperative, intraoperative, discharge and 30 days after discharge. A total of 68 variables were collected by patient, and follow-up was performed by telephone. Data completion and veracity were controlled by registry governance and administration. CC was calculated by the Crockcroft-Gault equation for estimation of glomerular filtration rate using SC, age, sex, and body weight.

EuroSCORE II values used in REPLICCAR is calculated on the website http://www.euroscore.org/calc.html. Outcome measures were hospital morbidity and mortality in the period from surgery to evaluation at 30 days, or to hospital discharge. Morbidity included severe acute renal failure (sARF), stroke and acute myocardial infarction (AMI).

### Statistical analysis

Continuous variables were expressed as mean ± standard deviation and categorical variables as percentages. Fisher exact test was used for contingency tables. Calibration was calculated by the Hosmer Lemeshow test, indicating that the model was adequately adjusted when p > 0.05. In the calibration of CC and SC, we analyzed the difference between expected and observed mortality and morbidity by nonlinear least squares (NLS). Therefore, a positive NLS indicates that the outcomes were better than expected. In addition to NLS, we evaluated the adjusted rate between observed and expected outcomes, the ´risk adjusted mortality quotient` (RAMQ). A RAMQ lower than 1 suggests that surgical outcome was better than the average outcome. CC and SC accuracy was analyzed by the area under the ROC curve. Using multiple logistic regression analysis, two multivariate models were built for mortality and two multivariate models were built for morbidity, one model using the variable CC, and the other using the variable SC. Regression analysis was performed by the stepwise selection method. Models with the dichotomous variable CC < 55mL/min were also tested. A P value < 5% was considered significant. Statistical analysis was performed using the SPSS desktop statistical software, version 22.0 for Windows (IBM Corporation Armonk, New York).

### Ethics and Consent form

This work was approved as a subproject of the online registry number 9696 of the Ethics Commission for Research Project Analysis (CAPPesq) of HCFMUSP, entitled “Heart surgery programs innovation using surgical risk stratification at the São Paulo State Public healthcare system: SP-Score-SUS study”.

## Results

### Subjects

Of 3,285 patients, 224 patients (6.8%) died and 263 (7.9%) had some morbidity. Mean age was 60.47 ± 12.3 years, and 1,195 (36.3%) were women. Mean body mass index was 26.7 ± 4.5 kg/m^2^. Reoperations were performed in 399 (12.1%) patients. A total of 1,428 (43.4%) patients with functional class III-IV and 1,180 (35.8%) emergency patients underwent surgery. Mean ejection fraction was 58.3 ± 11.2%. Mean SC and CC values were 1.25 ± 1.1 mg/dL and 72.6 ± 29.5 mL/min, respectively. Mean EuroSCORE II was 2.6 ± 4.3. A total of 1,862 (56.7%) MRS alone, 1,065 (32.4%) valve surgery alone and 358 (10.9%) MRS combined with valve surgery was performed.

### Association between SC and mortality

There was an association between SC and mortality (p = 0.0003). However, the model with SC subgroups did not adjust well for mortality in the Hosmer-Lemeshow test (H-L, p < 0.0001), Table 1.

**Table 1:** Expected mortality (EM) by serum creatinine adjusted for observed mortality (OM)

Serum creatinine	Cases	%	OM	EM	RAMQ (OM/EM)
< 0.80	341	10.4	15	20.96	0.72
0.80-0.87	346	10.5	16	21.72	0.74
0.88-0.93	310	9.4	9	19.69	0.46
0.94-0.99	235	7.1	10	15.07	0.66
1.00-1.03	322	9.8	19	20.78	0.91
1.04-1.10	350	10.6	16	22.83	0.70
1.11-1.20	381	11.6	22	25.2	0.87
1.21-1.34	325	9.9	21	21.87	0.96
1.35-1.59	319	9.7	28	22.02	1.27
≥ 1.60	364	11.1	68	33.86	2.01
Total	3293	100,0	224	224	

RAMQ: Risk Adjusted mortality quotient.

Our results showed that, although expected mortality by SC was associated with observed mortality in our sample, when SC was ≥ 1.60, expected mortality by the variable became significantly disproportionate (RAMQ > 2), underestimating the observed mortality. On the other hand, there is a similar number of patients between the groups (see supplementary figure A), which confirms the disproportion between OM and EM for higher SC levels.

### Association between creatinine clearance and mortality

There was a significant association between CC and mortality (p < 0.0001) and the model with CC subgroups adjusted well in the Hosmer-Lemeshow mortality test (H-L, p = 0.277), Table 2.

**Table 2:** Expected mortality (EM) by creatinine clearance adjusted for observed mortality (OM)

Creatinine clearance	Cases	%	OM	EM	RAMQ (OM/EM)
≥ 109	333	10.1	5	3.14	1.59
95-108	339	10.3	9	7.14	1.26
85-94	343	10.4	11	10.31	1.07
77-84	310	9.4	13	12.26	1.06
70-76	328	10.0	6	16.13	0.37
64-69	319	9.7	20	19.3	1.04
57-63	333	10.1	24	24.52	0.98
49-56	341	10.4	34	31.17	1.09
39-48	323	9.8	34	37.86	0.90
< 38	324	9.8	68	62.17	1.09
Total	3293	100.0	224	224	

RAMQ: Risk Adjusted mortality quotient.

In calibration, using creatinine clearance as predictive variable of the groups formed by the Hosmer Lemeshow test, there was no significant difference between expected mortality by CC and observed mortality (p = 0,277). Also, there is a similar number of patients between the groups (see supplementary figure B) that confirms that CC is a good predictor of mortality.

**Figure 1:**
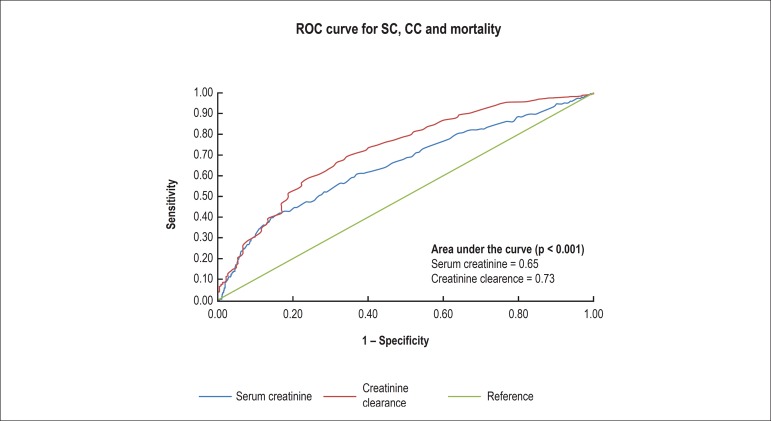
ROC curve for serum creatinine, creatinine clearance and mortality.

Analysis of the ROC curve (Figure 1), which measures the accuracy of the variable in discriminating between patients who died and those who survived, revealed that, when SC was used as predictive variable, the accuracy of the model was 0.65. However, when CC was used as predictive variable, the accuracy of the model in predicting mortality reached 0.73 (p < 0.001).

### Association between SC and morbidity (stroke, AMI, sARF)

There was an association between SC and morbidity (p < 0.0001). However, the model with SC subgroups did not adjust well to morbidity in the Hosmer-Lemeshow test (H-L, p < 0.0001), Table 3.

**Table 3:** Expected morbidity by serum creatinine adjusted for observed morbidity

		morbi = 1		morbi = 0	
Group	Total	Observed	Expected	Observed	Expected
1	341	13	23.80	328	317.20
2	346	14	24.83	332	321.17
3	310	13	22.59	297	287.41
4	235	16	17.34	219	217.66
5	322	14	23.95	308	298.05
6	350	23	26.40	327	323.60
7	381	20	29.26	361	351.74
8	325	32	25.53	293	299.47
9	319	32	25.90	287	293.10
10	364	86	43.42	278	320.58

RAMQ: Risk Adjusted mortality quotient.

Although we observed an association between expected morbidity by SC and observed morbidity in the sample, calibration by Hosmer-Lemeshow test showed a significant difference between expected mortality by SC and observed mortality in the groups.

### Association between CC and morbidity (stroke, AMI, sARF)

There was an association of CC with morbidity (p < 0.0001). CC subgroups adjusted well to morbidity in the Hosmer-Lemeshow test (H-L, p < 0,346), Table 4.

**Table 4:** Expected morbidity by creatinine clearance adjusted for observed morbidity

		morbi = 1		morbi = 0	
Group	Total	Observed	Expected	Observed	Expected
1	333	7	5.30	326	327.70
2	339	12	10.68	327	328.32
3	343	16	14.52	327	328.48
4	310	18	16.47	292	293.53
5	328	19	20.86	310	307.14
6	319	17	24.08	302	294.92
7	333	21	29.56	312	303.44
8	341	39	36.18	302	304.82
9	323	41	42.06	282	280.94
10	324	74	63.30	250	260.70

In addition to the association between expected morbidity by CC and observed morbidity in the sample, calibration by the Hosmer-Lemeshow test showed that there was no significant difference between expected mortality by CC and observed mortality in the groups.

**Figure 2:**
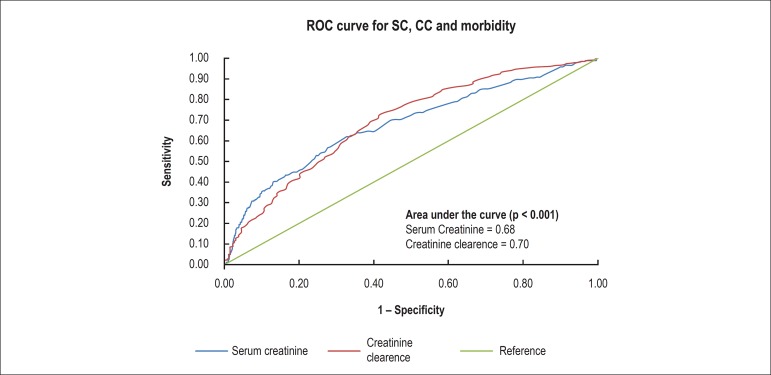
ROC curve for serum creatinine, creatinine clearance and morbidity.

Analysis of the ROC curve (Figure 2) showed that, when SC was used as predictive variable, accuracy of the model was 0.68 only. Nevertheless, when CC was used as predictive variable, accuracy of the model to predict observed mortality was 0.70 (p < 0,001).

### Multivariate model for mortality

In the upper part of the Table 5, we can see that when a multivariate model for mortality without CC was generated, the independent predicting variables were age, hematocrit, pulmonary artery pressure, type of hospitalization and functional class, but not SC. However, in the lower part of the table, when we created a multivariate model without SC, CC was in the model, achieving an accuracy of 0.768.

**Table 5:** Multivariate model for mortality

**Without Creatinine clearance:**			
		**Confidence interval 95%**	
**Effect**	**Risk estimation**	**Lower limit**	**Highest Limit**
Age	1.047	1.028	1.066
Hematocrit	0.924	0.891	0.958
Pulmonary artery pressure	1.020	1.008	1.032
Urgency Emergency	2.341	1.518	3.611
Functional class III/IV	2.136	1.063	4.292
**Accuracy = 0.762**			
**Multivariate model for mortality without Serum creatinine: **			
		**Confidence interval 95%**	
**Effect**	**Risk estimation**	**Lower limit**	**Highest Limit**
Age	1.038	1.019	1.058
Hematocrit	0.935	0.900	0.971
Pulmonary artery pressure	1.018	1.006	1.030
Creatinine clearance	0.989	0.978	0.999
Urgency Emergency	2.163	1.393	3.358
Functional class III/IV	2.087	1.037	4.198
**Accuracy = 0.768**			

### Multivariate model for morbidity

We can see in the upper part of the Table 6 that, when we created a multivariate model for morbidity without CC, the independent predicting variables were age, hematocrit, and SC, achieving an accuracy of 0.68. However, in the lower part of the table, it is shown that when we created a multivariate model without SC, CC was in the model, achieving an accuracy of 0.70.

**Table 6:** Multivariate model for morbidity

**Without Creatinine clearance:**			
		**Confidence interval 95%**	
**Effect**	**Risk estimation**	**Lower limit**	**Highest Limit**
Age	1.028	1.011	1.046
Hematocrit	0.940	0.908	0.973
Serum creatinine	1.127	1.018	1.240
**Accuracy = 0.68**			
**Modelo multivariado para morbidade sem Serum Creatinine:**			
		**Confidence interval 95%**	
**Effect**	**Risk estimation**	**Lower limit**	**Highest Limit**
Creatinine clearance	0.971	0.962	0.980
**Accuracy = 0.70**			

## Discussion

‘In patients undergoing cardiac surgery, renal function has an influence on mortality prediction.^2^ Many preoperative risk predictive models in patients undergoing cardiac surgery have confirmed the importance of renal function as a mortality predictor. In these models, ARF, necessity of dialysis and SC, used as categorical variables, are considered risk factors.

SC levels are affected by numerous factors that are independent of glomerular filtration rate: tubular secretion and reabsorption, endogenous production, irregular diet, extrarenal elimination, laboratory diagnostic techniques, and medications.^15,16^ Since assessment of renal function based on SC is associated with several limitations,^16,17^ and measurement of urinary CC takes a long time, many equations to estimate glomerular filtration rate using SC, body weight, age, sex and ethnic characteristics have been developed. All these equations, however, exhibit some limitations.

The most frequently used method to assess renal function in Medicare and in the national transplant waiting list in the US^18^ is the Cockcroft-Gault formula. This formula is not absolutely precise (e.g. in elderly patients) and may either overestimate or underestimate the renal function.^15,19^ Many studies on heart and renal failure showed a good correlation between CC estimated by the Cockcroft-Gault formula and the glomerular filtration rate.^20,21^ Due to its wide acceptance, this formula was chosen to be used in REPLICCAR.

It is worth mentioning that we performed binary analysis of CC (< 55 mL/min), which did not show any difference in comparison with continuous analysis of the variable. Nevertheless, in patients with SC ≥ 1.35 mg/dL, observed mortality was greater than expected mortality, reaching values two times greater than in patients with SC ≥ 1.60 mg/dL. Although SC has been used by Brazilian health care centers,^22,23^ even as a criteria of ARF stage classification,^24^ it should be analyzed with caution due to its lack of calibration in predicting mortality. This should start with the inclusion of CC in local risk scores, in which SC is still used as a binary data.

CC had greater predictive power for both mortality and morbidity than SC, assessed by the area under the ROC curve. However, there are difficulties in detecting differences between the variables by analysis of the standard deviation of the ROC curve. To address this issue, we constructed multivariate models by multiple regression to first evaluate the influence of CC on other variables, and then the influence of SC. In mortality model, regression analysis showed that when CC was excluded, SC was not an independent predicting variable, which suggests its inefficacy in this analysis. On the other hand, when SC was excluded, CC was not only an independent predicting variable, but also the only predictor in this model. This reinforces the importance of CC in the preoperative assessment, which has also been demonstrated in other studies performed in Brazil.^24^ Therefore, local models should also follow the tendency to include CC, similar to international scores.

Estimation of expected morbidity and mortality by the risk models, as well as their relationship with observed morbidity and mortality using NLS and RAMQ, represent effective analytical tools in the assessment of potential influence on morbidity and mortality (e.g. in detecting diseases in the preoperative period, choosing the type of surgery etc.).

CC, which is currently considered in EuroSCORE II, even as categories, has already been included in REPLICCAR as continuous variable and undoubtedly should be included in future risk models developed in Brazil. Therefore, there should be a preference for the use of CC, calculated by the Cockcroft-Gault equation over SC in the preoperative assessment of renal function.

The only clear limitation of this study is the fact that this was not a randomized study, which could specifically evaluate the impact of each variable. Although prospective registry is the most robust method for this type of analysis, it is worth to note that these results should be validated before being applied in other types of procedures and populations, as in pediatric population.

## Conclusion

This study shows that SC values greater than 1.6 underestimate the risk of hospital morbidity and mortality in patients undergoing coronary and/or valve surgery in Sao Paulo state. We encourage the calculation of CC for a more accurate, individualized assessment of renal function, aiming a better planning and optimization of perioperative care.

## Figures and Tables

**Supplementary Figure A:**
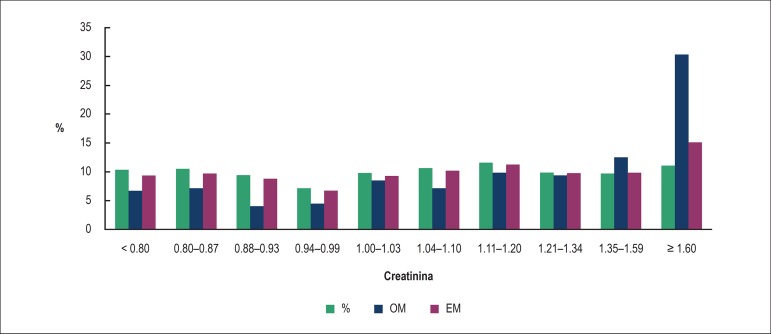
Percentage of patients, observed mortality (OM) and expected mortality (EM) for serum creatinine groups.

**Supplementary Figure B:**
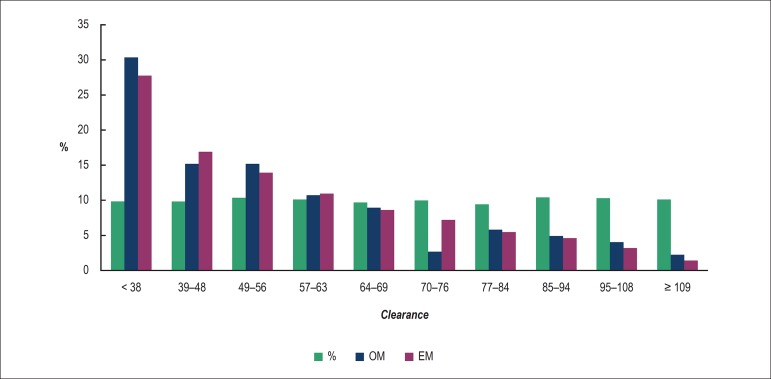
Percentage of patients, observed mortality (OM) and expected mortality (EM) for serum creatinine clearance groups.
